# Integrated Malaria Control in Madagascar: From Vector Dynamics to Community Interventions

**DOI:** 10.4269/ajtmh.25-0738

**Published:** 2025-12-23

**Authors:** Stéphanie Ramboarina, Laurent Kapesa, Jocelyn Razafindrakoto, Voahangy Rasolofo, Marie-Chrystine Solofoharivelo

**Affiliations:** ^1^ Scientific Directorate, Institut Pasteur de Madagascar, Antananarivo, Madagascar;; ^2^ U.S. President’s Malaria Initiative, USAID, Antananarivo, Madagascar

Malaria remains a persistent global scourge that now predominantly affects Africa. After years of progress, the disease is resurging across many regions of the continent, driven by lagging control efforts, COVID-19 disruptions, population movements, climate change, and land-use changes. Madagascar, an island nation off Africa’s southeast coast with an estimated 30 million inhabitants in 2024, has not been spared. It is among the African countries with increasing malaria incidence, including an estimated 71% rise between 2022 and 2023.^1^

Since 2002, Madagascar has achieved notable progress in malaria control through coordinated efforts led by the Ministry of Public Health and its partners to improve prevention and treatment measures, resulting in substantial reduction in the national malaria burden.^2^ However, localized resurgences have been reported since 2017, driven by structural weaknesses in the health system, environmental pressures such as flooding and climate change influencing mosquito behavior, and social factors including seasonal migrations and growing insecurity that forces populations to sleep outdoors or avoid health centers.^3^^–^^6^ In addition, COVID-19 significantly reduced malaria service utilization, undermining control efforts. By 2023, Madagascar accounted for approximately 2.5% of global malaria cases and deaths.^1^

The national malaria strategic plan for 2018–2022 envisioned “a country without malaria” structured around several strategic axes.^7^ These included strengthening case management, particularly at the community level, reinforcing preventive measures such as integrated vector management, long-lasting insecticide nets (LLINs), indoor residual spraying (IRS), and chemoprevention, and enhancing surveillance and response capacity. Routine District Health Information System 2 data showed a marked rise in malaria incidence in Madagascar, increasing from 36.7 to 82 cases per 1,000 population between 2018 and 2021. Over the same period, proportional morbidity, the proportion of outpatient consultations due to malaria, increased from 9.3% to 22.8% (Ministry of Health Madagascar. District Health Information Software 2 (DHIS2): Routine malaria data, 2018–2021: unpublished dataset). Yet, reported mortality declined by 41% (from 927 to 547 deaths) with malaria accounting for only 0.4% of recorded deaths in 2021. This likely reflects better case management, wider access to treatment, and improved surveillance of uncomplicated cases.

Ministry of Public Health malaria control efforts were strongly supported by technical and financial partners. Since 2008, the United States Agency for International Development (USAID), through the President’s Malaria Initiative, had been a major contributor to Madagascar’s malaria control program, although this support has since been substantially cut. From 2019–2024, USAID funded the Research, Implementation, Surveillance, and Evaluation (RISE) project, implemented by the Institut Pasteur of Madagascar. This 5-year project aimed to strengthen data-driven decision making by the Ministry of Public Health and included operational research, evaluation studies, malaria surveillance activities, and targeted responses aligned with the national strategic plan.

This supplement to the *American Journal of Tropical Medicine and Hygiene* comprises six articles reporting findings from activities conducted in the RISE project and other malaria research projects in Madagascar. Five original papers focus on community-based malaria management, antimalarial treatment efficacy, and vector control, while a sixth article provides a systematic review of malaria vectors in Madagascar, offering an integrated overview of the country’s evolving malaria epidemiology.

Between 2019 to 2022, multiple field studies were conducted through the RISE project to strengthen prevention, control, and case management. These included a malaria community case management (mCCM) study in Farafangana district (2019–2021) in the southeastern part of Madagascar, two therapeutic efficacy studies across four sentinel sites (2020 and 2022), and bioefficacy evaluations of LLINs distributed during the 2018 and 2021 national campaigns.

Community engagement is central to malaria control, yet rural populations, the most affected by malaria, face geographic, environmental, and financial barriers to accessing care. Community Health Workers (CHWs) generally focus on children under five years of age, leaving older children and adults underserved. Addressing this gap, the first paper of the collection presents qualitative findings from a cluster-randomized trial (November 2020 - December 2021) assessing the feasibility and acceptability of expanding mCCM to all age groups in Farafangana district.^8^^,^^9^ The approach was well received by the population and healthcare workers, was associated with improved malaria knowledge, and strengthened CHW roles, although structural and community barriers, particularly high treatment costs, persist.

Integrating age-expanded mCCM into national strategies is crucial to ensuring timely diagnosis and treatment while ensuring the continuum of care across all ages. Regular therapeutic efficacy monitoring quantifies first-line drug efficacy, detects early signs of emerging resistance, and underpins public confidence in malaria services. Madagascar has consistently demonstrated high antimalarial efficacy, with minimal drug resistance detected to date. The second paper in this collection demonstrates that artesunate–amodiaquine and artemether–lumefantrine remained highly effective and safe for treating uncomplicated *Plasmodium falciparum* infection in 2020, with cure rates exceeding 97%.^10^ A subsequent survey conducted in 2022 corroborated these results, supporting continued use of these first-line regimens and the need for ongoing resistance monitoring.

Targeted vector control remains a pillar of malaria management in Madagascar, where the majority of the population lives in endemic areas. Despite widespread LLIN and IRS deployment, recent outbreaks highlight key limitations. In the third paper, Nepomichene et al. report a rapid decline in the bioefficacy of LLINs distributed in 2018, after just one year of use, falling well below the expected 36-month lifespan of these products.^11^ These results suggest that improvements in net quality, storage conditions, and user practices are urgently needed. Complementary research on IRS is presented in the fourth paper by Nepomichene et al., demonstrating that SumiShield^®^ 50WG, a clothianidin-based insecticide, maintains high mosquito-killing efficacy for up to eight months, effectively covering Madagascar’s transmission season and sustaining the impact of vector control interventions.^12^

Despite these results, available interventions are increasingly threatened by Madagascar’s high vulnerability to climatic, environmental, and human-driven disturbances, which are reshaping mosquito distribution and behavior and undermining the effectiveness of vector control efforts. Recent studies reveal variability in mosquito density, species diversity, biting preferences, vector habitats, and *Plasmodium* infection rates across the five bioclimatic zones of the country, influenced by humidity, aridity, and seasonal changes. This has shifted vector composition, with secondary vectors increasingly driving transmission. While *Anopheles funestus, An. arabiensis*, and *An. gambiae s.s*. remain the primary vectors, *An. coustani* is now widespread, and *An. squamosus/cydippis* is emerging in the southeast.^13^^–^^15^ The growing prominence of secondary vectors underscores the need for further research to fully understand nationwide malaria vector ecology and transmission dynamics.

In this context, Goupeyou et al. provide a systematic review of *An. mascarensis*, the primary vector in eastern and southeastern regions of Madagascar, which is considered a secondary vector in other regions.^16^ The authors highlight marked behavioral and ecological differences between eastern and highland populations, suggesting that *An. mascarensis* may represent a complex of sibling species. Advances in molecular tools offer opportunities to investigate this hypothesis and clarify the epidemiological importance of *An. mascariensis* in malaria transmission in Madagascar. Importantly, unlike primary vectors such as *An. gambiae* and *An. arabiensis*, which are increasingly resistant to insecticides, *An. mascarensis* remains fully susceptible, reinforcing its relevance for targeted vector control.^12^

Persistent malaria outbreaks following LLIN and IRS campaigns emphasize the need to integrate parasitological and entomological surveys to understand Madagascar’s evolving malaria landscape. All four major human *Plasmodium* species (*P. falciparum*, *P. vivax*, *P. malariae*, and *P. ovale*) are known to circulate in the country, with *P. vivax* increasing in prevalence. Many *P. vivax* infections are likely subclinical or misdiagnosed as *P. falciparum* due to similar symptoms and limitations of rapid diagnostic tests. Understanding how different vectors contribute to transmission of these parasites remains a key challenge for malaria control and elimination. The sixth paper in the collection presents an integrated outbreak investigation in the endemic district of Farafangana shortly after LLIN distribution and IRS campaigns in 2018.^17^ Multiple vector species were identified including *An. coustani, An. gambiae s.s.*, *An. funestus*, and *An. mascarensis*, with many exhibiting predominantly outdoor biting behavior and contributing to ongoing transmission. All infected mosquitoes carried *P. falciparum*. More than 30% of surveyed individuals were infected, most of them asymptomatic children, highlighting substantial hidden transmission. These findings underscore the urgent need for vector control strategies targeting outdoor-biting mosquitoes alongside existing indoor interventions.

Despite enhanced, context-specific interventions over the past decade, Madagascar remains off track to control or eliminate malaria. Transmission is highly heterogeneous across regions, districts, communes, and fokontany (the smallest administrative unit in Madagascar, similar to a village), and shaped by intertwined climatic, socioeconomic, and cultural factors that create a stratified epidemiological landscape. Lessons from recent surveys were incorporated into the 2023–2027 National Strategic Plan, which promotes stratum-specific strategies to improve access to care, strengthen health education, optimize coverage, and link prevention with rapid outbreak response. Importantly, Madagascar has so far not reported drug resistance, while the increasing threat of insecticide resistance lurks and complicates malaria control globally. New approaches such as larvicidal interventions therefore warrant further exploration. Continued efforts to improve malaria case management across the country’s five epidemiological zones are essential to enable timely responses and to guide future strategies in near real time.^3^

The resurgence of malaria in Madagascar highlights the limitations of donor-driven, one-size-fits-all interventions, and the fragility of national programs as external funding declines. A paradigm shift is urgently needed: from externally led efforts to locally anchored, context specific strategies tailored to Madagascar and informed by updated stratification in the 2023–2027 national strategic plan for malaria control. This shift requires strategic partnerships, integrated community-level programs, and the empowerment of local health workers, national stakeholders, and communities to lead planning, implementation, and monitoring. Such a shift must be supported by a strong government commitment to strengthen domestic resource mobilization, including increasing the national budget for health and education and efficient use of these limited resources to ensure that local priorities are addressed. External partners remain essential, but their role must evolve toward reinforcing national ownership rather than setting the agenda. Sustainable malaria control in Madagascar hinges less on the scale of external funding and more on the ability of local actors to design and sustain context-specific and cost-effective interventions that reflect the island’s unique epidemiological and social realities.
